# Efficient Simulation of the Spatial Transmission Dynamics of Influenza

**DOI:** 10.1371/journal.pone.0013292

**Published:** 2010-11-04

**Authors:** Meng-Tsung Tsai, Tsurng-Chen Chern, Jen-Hsiang Chuang, Chih-Wen Hsueh, Hsu-Sung Kuo, Churn-Jung Liau, Steven Riley, Bing-Jie Shen, Chih-Hao Shen, Da-Wei Wang, Tsan-Sheng Hsu

**Affiliations:** 1 Institute of Information Science, Academia Sinica, Taipei, Taiwan; 2 Epidemic Intelligence Center, Centers for Disease Control, Taipei, Taiwan; 3 Department of Computer Science and Information Engineering, National Taiwan University, Taipei, Taiwan; 4 Centers for Disease Control, Taipei, Taiwan; 5 Department of Infectious Disease Epidemiology, University of Hong Kong, Hong Kong; 6 Department of Radiation Oncology, Far Eastern Memorial Hospital, Taipei, Taiwan; 7 Department of Computer Science, University of Virginia, Charlottesville, Virginia, United States of America; Dana-Farber Cancer Institute, United States of America

## Abstract

Early data from the 2009 H1N1 pandemic (H1N1pdm) suggest that previous studies over-estimated the within-country rate of spatial spread of pandemic influenza. As large spatially resolved data sets are constructed, the need for efficient simulation code with which to investigate the spatial patterns of the pandemic becomes clear. Here, we present a significant improvement to the efficiency of an individual-based stochastic disease simulation framework commonly used in multiple previous studies. We quantify the efficiency of the revised algorithm and present an alternative parameterization of the model in terms of the basic reproductive number. We apply the model to the population of Taiwan and demonstrate how the location of the initial seed can influence spatial incidence profiles and the overall spread of the epidemic. Differences in incidence are driven by the relative connectivity of alternate seed locations. The ability to perform efficient simulation allows us to run a batch of simulations and take account of their average in real time. The averaged data are stable and can be used to differentiate spreading patterns that are not readily seen by only conducting a few runs.

## Introduction

The current global spread of a novel influenza strain [1] highlights gaps in our understanding of the spatial component of disease transmission at national and regional scales. For example, the early summer 2009 wave in the United States affected some populations much more so than others (Centers for Disease Control, USA), even at similar latitudes. In addition, there was substantial transmission in parts of southern England throughout the summer of 2009, but very little in most of northern mainland Europe (European Centre for Disease Prevention and Control). This slow progression between national and regional level synchrony is not obviously consistent with previous theoretical studies of the within-country dynamics of pandemic influenza [2]–[4], in which census-reported commuting patterns and airline flight data were used to characterize very rapid spatial spread. Explaining these early patterns of spatial spread for the 2009 pandemic will likely be an active area of epidemiological research in the coming years.

Stochastic spatial transmission models, in which individuals or small communities are represented explicitly in space, are an extension of more traditional approaches and have been a valuable tool in the study of infectious diseases in humans and animals [5]. Traditionally, mathematical models of epidemics often take the form of deterministic differential equations in which the variables represent the expected number of individuals in broad disease classes (e.g., susceptible, infected, or recovered) [6]. Although such models can be extended to model the geographic spread of infectious diseases on patches [7], when it is not clear which spatial scales are most important, it is difficult to use compartmental approaches with confidence.

Here, we describe an algorithmic refinement of a spatial stochastic model of individuals and their communities. This framework was originally designed to investigate community interventions against influenza in a generic sense [8]. It was later extended to examine the optimal response to a bio-terrorist smallpox attack [9] and to examine the potential for the containment of influenza pandemic in large well-mixed populations [10]. A spatial component was added to the model to study the feasibility of containing an emergent influenza pandemic in a rural setting in Southeast Asia [11]. In its last major development, the underlying algorithm was parallelized to allow it to run with a population of 300 million, and used to predict the likely impact of mitigation measures on an influenza pandemic in the United States [2]. More recently, the same framework has been used to describe the likely fall wave transmission dynamics for H1N1pdm in Los Angeles County [12], and to study the effects of school closure strategies in Allegheny County, Pennsylvania [13].

We have implemented a more efficient algorithm for this popular disease transmission model. We demonstrate increased computational efficiency compared with previous implementations and we describe a parameterization scheme for the model using the basic reproductive number, rather than the per contact transmission potential. We illustrate the utility of the refined model with simulation studies of seeding dynamics for a pandemic of influenza in Taiwan.

## Materials and Methods

Our model incorporates epidemiological attributes of viral infection with computer generated mock population to simulate the spatio-temporal spreading of pandemic influenza viruses. The mock population is constructed according to national demographics and daily commuter (worker flow) statistics from Taiwan Census 2000 Data (http://www.stat.gov.tw/) in order to retain some population characteristics. The model is, effectively, a highly connected network model representing the 23 million people living in Taiwan. The connection between any two individuals indicates the possibility of regular (daily) and relatively close contact that could result in the successful transmission of the flu virus. A contact group is a close association of individuals, where every member is connected to all other members in the group. We designate ten classes of such contact groups in our model: community, neighborhood, household cluster, household, work group, high school, middle school, elementary school, daycare center, and playgroup. It is important to note that these contact groups do not represent all people at any physical location such as a workplace or school, but rather the groups of people who share the same surrounding activities and sustain regular close contact for potential viral infection. Furthermore, the entire population is classified into five age groups: preschoolers (0–4 years old), school-age children (5–18 years old), young adults (19–29 years old), adults (30–64 years old), and elders (65+ years old). Each individual is a member of one of the five age groups throughout the simulation, and can belong to several contact groups simultaneously at any time. The probability of any two individuals staying in contact that could result in the successful transmission of the flu virus is called the contact probability, and an empiric value is assigned depending on the group where contact occurs and the ages of both individuals. Age not only affects the probability of an individual being infected, it also determines the individual's daytime contact groups: preschoolers stay either in daycare centers or in playgroups; school-age children stay either in schools or in households as dropouts; young adults and adults stay either in work groups or in households if unemployed. Each simulation runs in cycles of two 12-hour periods, daytime and nighttime, with each cycle representing a day in the simulation. The simulation can cover any specified duration of days; we usually operate in 180 days for typical influenza season, but there are times when 365 days duration is imperative for a slow progressing epidemic. Contact occurs between individuals in each contact group every day, there are no exceptions for weekends or holidays until we can properly ascertain their effects. During nighttime, contact occurs only in communities, neighborhoods, household clusters, and households; whereas in the daytime, contact occurs in all contact groups. Children do not go outside of their residential community for daytime activities because the probabilities for such occasional contacts are too low to be captured by any contact group. The only inter-community transmission occurs when working adults commute between household and work group as specified by worker flow data. The implementation details of the base model are provided in supporting text (Appendix S1); model parameters, such as the full listing of contact probabilities, are given in the supporting information of a study by Germann *et al.* [2]

The discrete-time simulation of infection events in individual-based epidemic models can be reduced to the generation of binomial deviates. Within any given model, there can be many types of infectious individual and many types of susceptible individual. For example, there can be many age groups and many stages in the natural history of a disease. The set of all possible pairs in which the first element is an infectious individual and the other element is a susceptible individual (an *I*–*S* pair) defines the set from which infection events can be simulated at any point in time. If many of the pairs have exactly the same probability of generating an infection (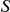 of exactly same type and 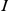 of the exactly the same type) then many infection events can be generated with relatively few binomial deviates. However, if the pairs are largely different, then many binomial deviates need to be drawn to generate a similar number of infections. The introduction of spatial dimensions into individual-based formulations greatly increases the heterogeneity of the model because every small group of individuals with a unique location forms, effectively, their own risk group.

A high-level description of a naive algorithm for the basic model is presented in Algorithm 1 (Table 1). The basic idea is to substantiate viral transmission to every susceptible individual in every contact group of every infectious individual during every 12-hours period of the simulation.

**Table 1:** Algorithm 1: Naive algorithm.

**foreach** *time period* 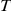 **do**
**foreach** *infected individual* 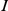 **do**
update the status of 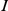 according to 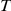
**if** 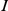 *is infectious* **then**
**foreach** *individual* 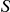 **do**
**if** 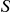 *is susceptible* **then**
**foreach** *contact group* 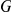 **do**
**if** 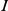 *and* 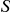 *are in the same group* 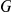 **then**
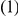 calculate the probability 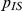 , that 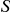 is infected by 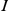
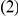 use a random number generator to decide whether 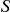 is infected by 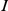 with a probability of 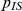
**if** 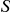 *is infected* **then**
update the status of 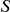
**end**
**end**
**end**
**end**
**end**
**end**
**end**
**end**

The Sieve algorithm we have developed greatly improves the efficiency with which infection events can be generated across large numbers of similar risk pairs. Here, we briefly describe the key features of the algorithm as it relates to the efficient simulation of spatial epidemics. The methods are described in more details elsewhere [14]. In essence, the approach is to use lazy evaluation for large groups of pairs with similar probabilities of an infection event. For example, one infectious individual a in community 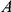 has a certain maximum probability of infecting members of community 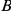, based on the flow of workers between those two communities. The precise probability of infection for each member of community 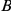 will depend on their age and other risk variables. However, the maximum probability for any individual in group 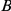, 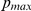, may be very small if the worker flow between 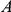 and 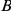 is small. Working with the Sieve algorithm, our first step is to generate a random variable for the provisional number of infection events that occur by assuming that all pairs have the same probability of an infection occurring. This however generates too many infections, and the second step is to select specific pairs at random and either accept or reject provisional infections using the precise probability of infection between individual 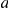 and each individual 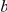 (in the provisional set of infections in community 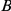). We define the precise probability to be 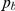. If we accept each provisional infection event with probability 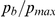, it is clear that the overall probability of individual 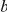 being infected is equal to 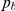. Therefore, our method reiterates the same stochastic process as if we evaluated each individual 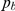 separately, and is not an approximation.

A high-level description of our improved algorithm is available in Tables 2 and 3.

**Table 2:** Algorithm 2: Our improved algorithm.

**foreach** *time period* 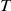 **do**
**foreach** *infected individual* 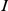 **do**
update the status of 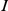 according to 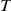
**if** 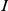 *is infectious* **then**
**foreach** *contact group* 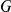 that 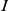 is in **do**
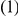 calculate the infection probabilities 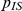 between 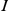 and all susceptible individual 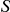 in 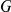
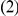 use the Sieve algorithm below to decide all individuals in 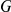 to be infected by 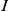
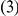 update the status of newly infected individuals
**end**
**end**
**end**
**end**

**Table 3:** Algorithm 3: Sieve algorithm.

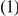 let 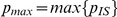 for all susceptible individual 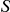 in 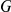
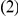 let 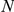 be the number of susceptible individuals in 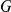
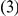 decide a tight bound 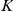 that is the upper bound of possible infected persons according to a binomial distribution with an inclusion probability 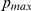 and 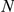 trials
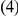 randomly pick 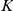 candidates from the group of susceptible individuals in 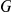
**foreach** *picked candidate* 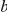 **do**
use a random number generator to decide whether 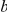 is infected by 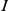 with a probability of 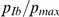
**end**

We are able to prove that the statistical behaviors of the Sieve algorithm are the same as the naive algorithm where each candidate is decided one by one, sequentially. The proof of this equivalence is given in [14]. Note that our Sieve algorithm decides a set of candidates in a batch. One of the reasons that our algorithm can run faster is because in practice, 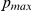 is very small. Thus, the size of the candidates 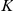 selected in the Sieve algorithm is much smaller than 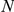, the pool of people to be considered.

By treating the model explicitly as a network, we calculate the average number of secondary cases *a priori*, rather than using semi-empirical methods to calibrate the model. The basic reproductive number 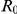 is the expected number of secondary infections generated by a single typically infectious individual in an otherwise susceptible population [15]. 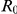 is a threshold parameter that determines whether an infectious disease will spread through a population. Strictly, for models with multiple types of infectious individuals, 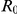 should be defined in terms of a next generation matrix and an eigenvector for the exponential phase of growth. The eigenvector is important in that it defines what is typical during the exponential phase. Often, a typical type of infectious individual will be different from a randomly chosen individual. For network models of infectious disease, the formal approach presents some problems because every individual is, essentially, a different type. Therefore, we follow many previous network models and use the average number of secondary cases per randomly chosen individual as 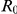.

**Figure 1:**
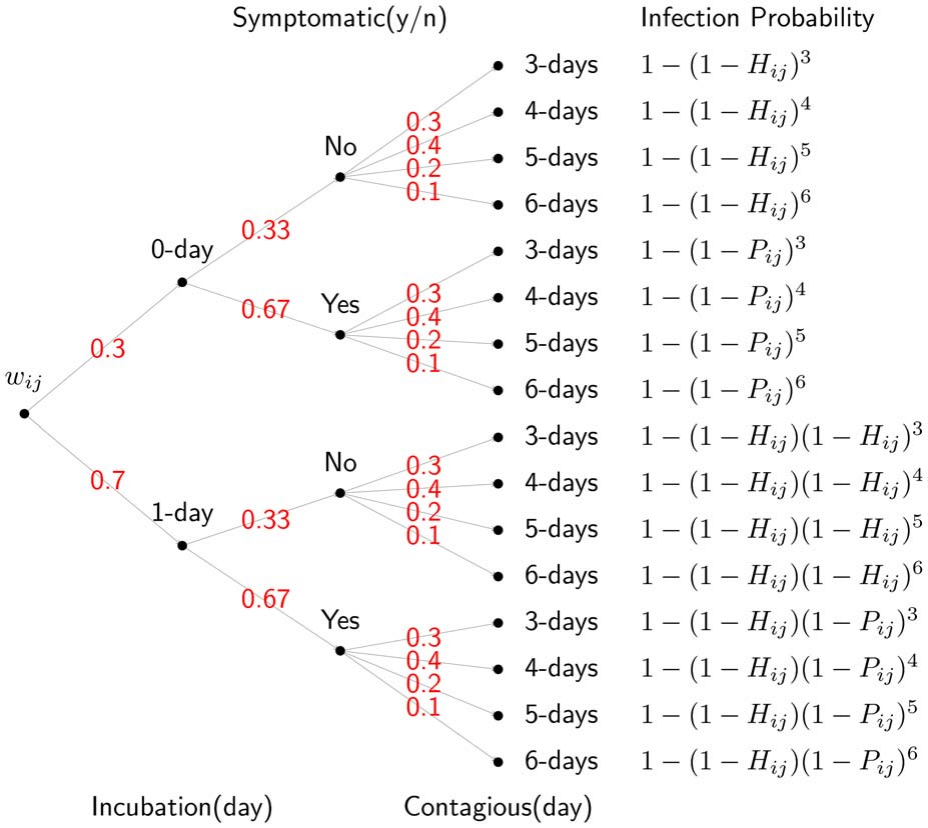
The computation of the probability that individual 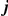 will be infected by individual 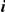 according to the natural history model.

Based on the influenza model and parameters, we compute the probability that infectious individual 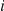 will infect susceptible individual 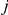, namely 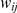, as follows. First, the infection probability resulting from 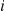 and 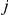
's contact in group 
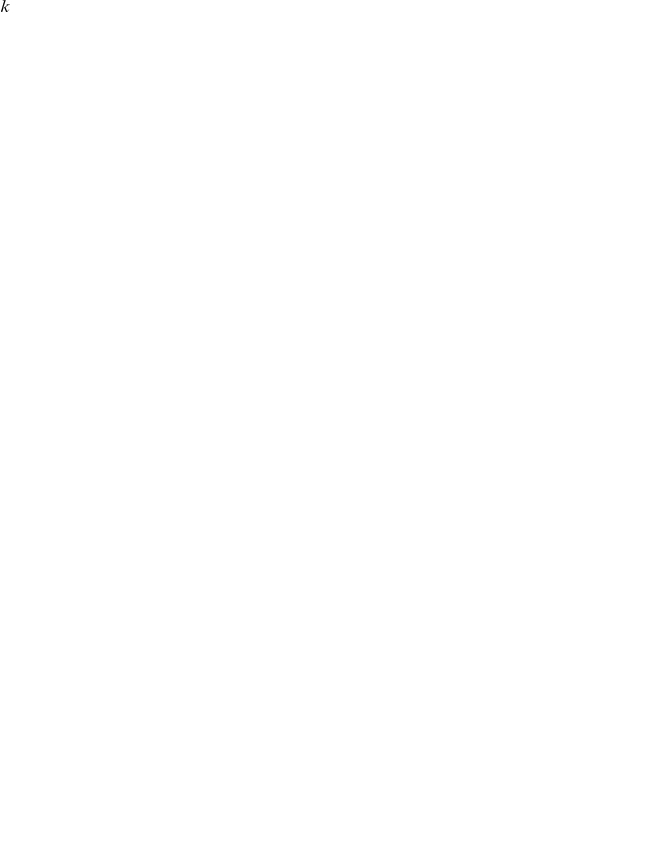
 is defined as 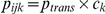, where 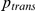 is the disease-dependent transmission probability and 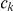 is the group-dependent contact probability. Second, 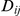 is the set of 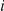 and 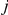
's contact groups in the daytime, and 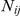 is the set of 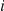 and 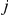
's contact groups during the night. The intersection of 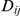 and 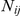 can be either empty or nonempty. Third, when the infectious individuals are incubating or asymptomatic, the infection probability is reduced by a factor of 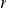, where 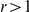. For clarity, we define 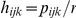. In our model, the current setting of 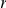 is two, as in [2]. Thus, in conjunction with all daytime and nighttime contacts, the daily infection probability is calculated by
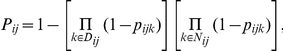


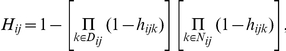
where 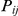 is the daily infection probability when individual 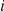 is symptomatic, and 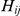 is the daily infection probability when individual 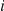 is incubating or asymptomatic. Finally, by adopting the natural history model, 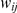 can be calculated as the weighted sum of all branches in Figure 1. The expected number of people infected by individual 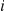 is 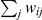, when 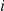 is the single infectious case in the otherwise susceptible population. Assuming that each individual has an equal chance of being the initial infectious case, we calculate the expected number of secondary infections for everyone in the entire population; and by definition, the Theoretical 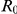 is the average of all such secondary infections. Table 4 lists the value of Theoretical 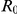 for a selected range of 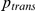, along with two 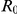 estimations derived from alternative methods. The first method samples, stratified by age group, 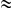
2,000 people as the index cases and calculates 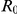 for the sample group. We then define the Sample 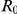 as the average 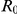 from 100 such sample groups. We find that even with a small sample size, the Sample 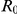 approximates the Theoretical 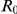 closely if we take sufficient samples. In addition, since the model population remains unchanged throughout the simulations, we can estimate 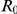 based on the prevalence of infections at the point of endemic equilibrium [16]. The second method is to average the estimated 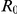 from 100 baseline simulations for each 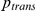, we call it the Simulated 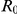. The estimated 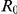 for each simulation result is calculated using the following formula
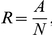


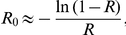
where 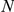 is the number of people in the population, 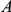 is the number of people who experience the event (become infected), and 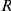 is the proportion of the population who become infected, also known as the infection attack rate.

**Table 4:** Comparison of 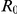.

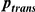	Theoretical 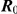	Sample 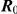	Simulated 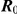
0.07	1.114	1.114 (1.133E-03)	1.147 (1.811E-04)
0.08	1.269	1.270 (1.407E-03)	1.262 (6.985E-05)
0.09	1.424	1.424 (1.468E-03)	1.379 (6.777E-05)
0.10	1.577	1.576 (1.558E-03)	1.500 (8.114E-05)
0.11	1.730	1.730 (1.796E-03)	1.622 (7.376E-05)
0.12	1.882	1.882 (2.101E-03)	1.745 (7.623E-05)
0.13	2.033	2.033 (2.154E-03)	1.868 (9.316E-05)
0.14	2.183	2.184 (2.577E-03)	1.990 (9.551E-05)
0.15	2.333	2.333 (2.538E-03)	2.111 (1.011E-04)
0.16	2.482	2.481 (2.808E-03)	2.231 (1.188E-04)
0.17	2.630	2.631 (2.916E-03)	2.349 (1.240E-04)
0.18	2.777	2.777 (2.664E-03)	2.466 (1.202E-04)

List of 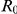, calculated by three different methods, for the selected range of 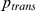. Theoretical 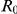 is the average number of expected secondary infections per individual in the entire population. Sample 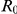 is the average of 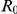 derived from 100 samples of 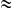
2,000 initial infectious case; the 95% confidence interval (CI) is listed in parentheses. Simulated 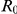 is the average of 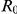 estimations derived from 100 baseline simulations; the 95% CI is listed in parentheses.

## Results

**Figure 2:**
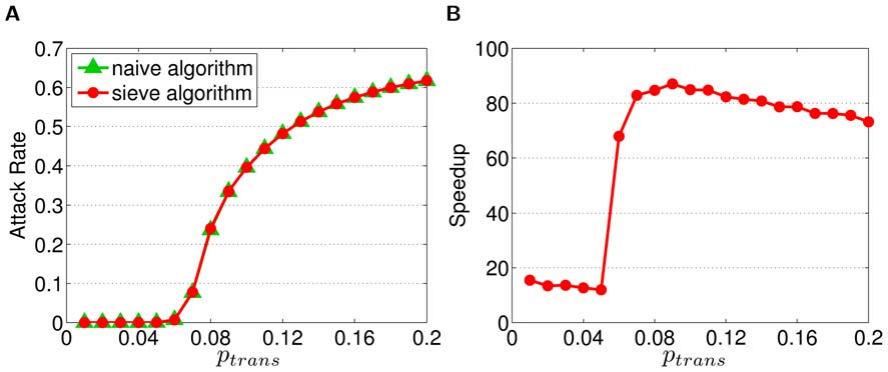
The precision and efficiency of the Sieve algorithm, as applied to a model of pandemic influenza transmission in Taiwan. (A) Demonstrates the correct implementation of the Sieve algorithm such that the attack rates from both algorithms stay nearly identical throughout the selected range of 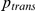. (B) Shows the speedup of the Sieve algorithm for the selected range of 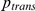. Speedup is defined as the ratio of the average computation time for the naive algorithm over the Sieve algorithm.

The Sieve algorithm shows significant improvement over the naive algorithm when applied to a real-world application. For a simulation involving population of 23 million people (approximately the size of Taiwan's population), we calibrated the strength of transmission to have an infection attack rate of 60% (a severe pandemic) and let the infectious period of an infector be, on average, three days. Even with a coarse half-day time step, the naive algorithm would still need to evaluate an order of 1,015 interactions (providing every infectious individual has a non-zero probability of infecting any susceptible host). By using the re-sampling approach of the Sieve algorithm, the execution time is drastically reduced (Figure 2B) without any notable loss of precision (Figure 2A).

**Figure 3:**
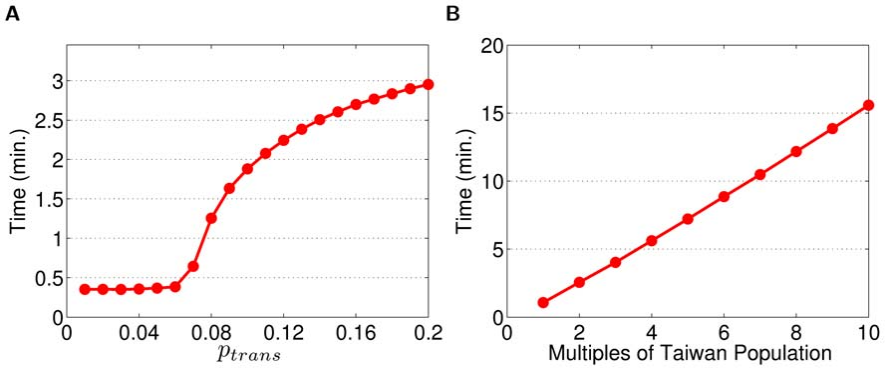
Average computation time for various 180-day baseline simulations. (A) Simulation time on a mock Taiwan population for the selected range of 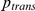. (B) Simulation time on multiples of Taiwan population for 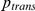 = 0.10.

These performance data were derived from groups of 32 runs of the baseline simulation for each 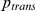 and algorithm combination. On a server with dual Intel Xeon W5580, quad-cores, 3.20 GHz CPUs and 48GB DDR3 memory, and 16 simulations running concurrently, the Sieve algorithm finishes 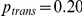 baseline simulation in just under three minutes (Figure 3A); in contrast: the naive algorithm takes about three hours and twelve minutes. Figure 3A illustrates the average simulation time of the Sieve algorithm, including 20 seconds for generating the mock population. The simulation time remains relatively low up to a threshold value (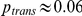), after which both the simulation time and cumulative number of infections (attack rate in Figure 2A) increase substantially. Figure 3B shows the time required for the simulation of multiples of Taiwan's population for 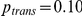, here we perform a single simulation for each population size due to memory limitations.

### Stochastic convergence

The stochastic process, by nature, involves non-deterministic trials evolving through time and abiding by miscellaneous characteristics with probability distributions. This means that even if all conditions are known in advance, there will be numerous possible outcomes, while some are more probable than others. With all trials guided by the same set of characteristics and probability distributions, the sequence of essentially random events is expected to settle into a pattern. Multiple realizations of the same scenario are necessary to elucidate this underlying pattern. A fast realization tool for the stochastic process is especially beneficial in dealing with various aspects of the model itself, such as sensitivity analysis.

**Figure 4:**
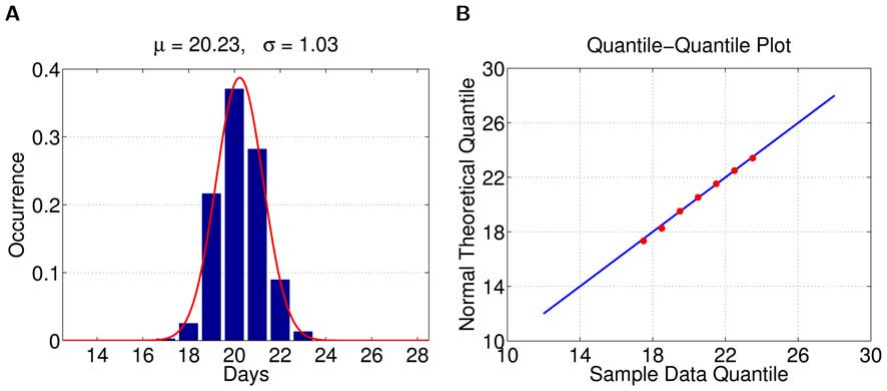
Statistical properties of simulation results. (A) Histogram and the estimated normal distribution for the average day of the 1,000-th symptomatic case. (B) Quantile-quantile (q-q) plot of the observed distribution with the theoretical normal distribution.

Next, we describe experiments conducted to assess the variability of the simulation results. First, we randomly picked a mock population and simulated 2,000 baseline realizations with constant transmission parameters. For each of the 2,000 realizations, we extracted information on important properties, such as the day of the 1,000-th (10,000-th, …) symptomatic case and the final number of infected people. We then treated the statistics from all 2,000 results as if they were the real sample space and assumed that the parameters of the real unknown sample space were comparable. Thus, each production run is merely a sample derived from the 2,000-run sample space (2KSS). First, we observe that the histograms of the important properties are all bell-shaped. We use a maximum likelihood heuristic to estimate the most likely normal distribution to match the histogram, as shown in Figure 4A. Next, we then compare the observed distribution with the theoretical normal distribution in a quantile-quantile (q-q) plot. The q-q plot is a graphical technique for determining if two data sets come from populations with a common distribution. It is a plot of the quantiles of the first data set against the quantiles of the second data set. By a quantile, we mean the fraction (or percentage) of points below a given value. A 45-degree reference line is also plotted. If the two sets come from a population with the same distribution, the points should fall approximately along this reference line [17]. As illustrated in Figure 4, the normality of the observed distribution is not only visually correlated on the left, and also statistically verifiable on the right.

We then assess the variability among groups of simulation results and attempt to establish an acceptable number of simulation runs that would represent all possible outcomes with high confidence. We calculate the 95% CI for selected important properties in groups of 
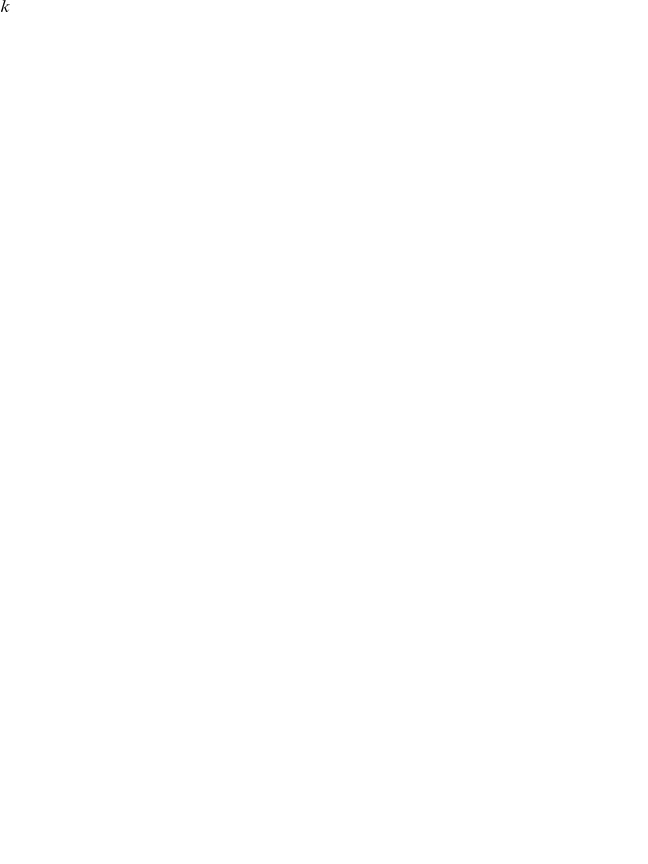
 simulation runs, where 
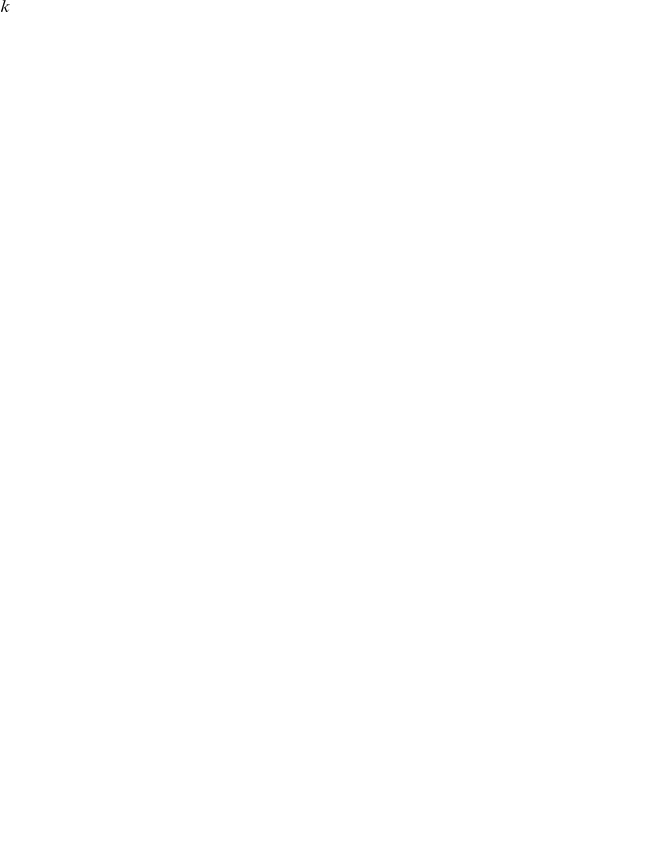
 ranges from 2 to 100. For each value of 
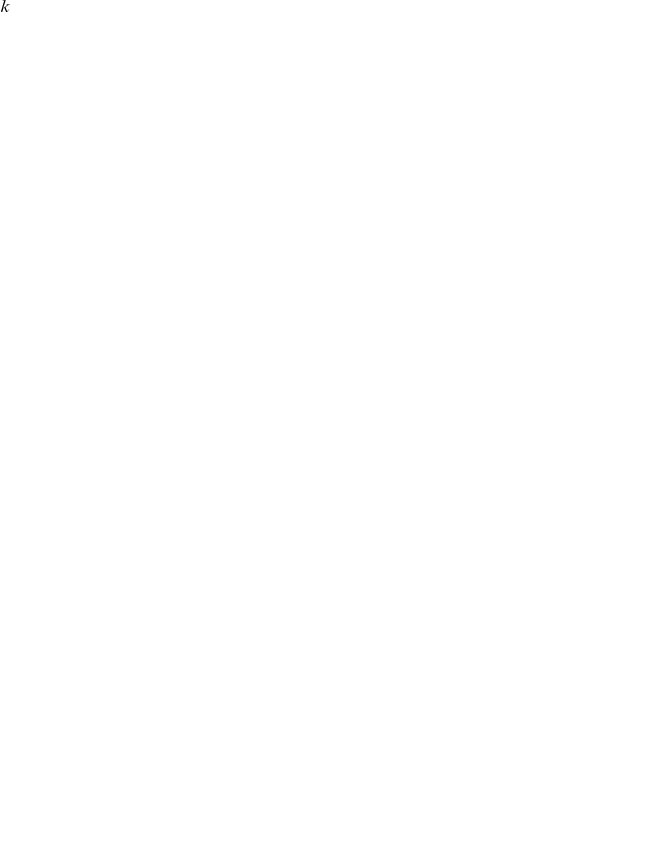
, we conduct 1,000 experiments by sampling 
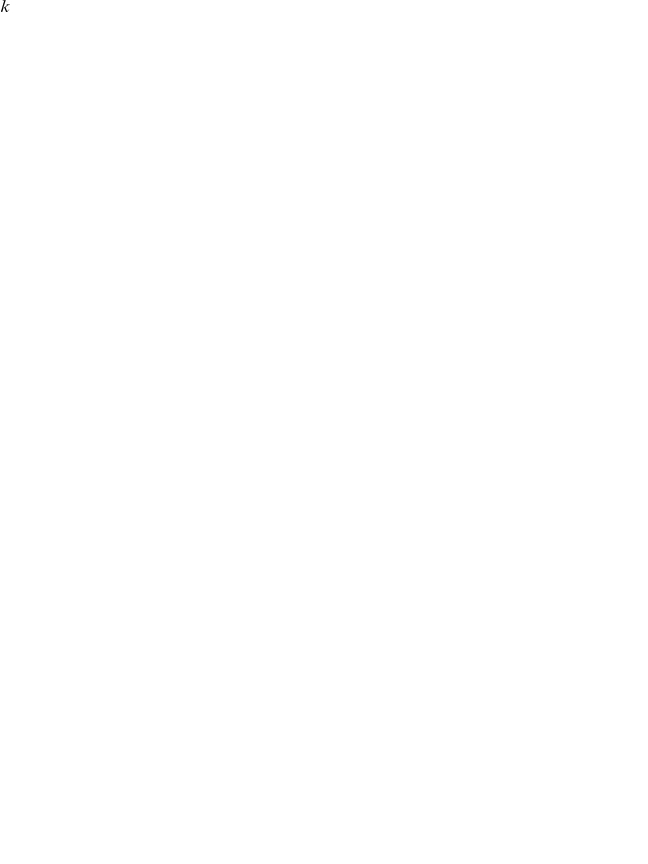
 instances out of 2KSS, and calculate the corresponding 95% CIs for each experiment. We then calculate the mean and standard deviation of 95% CIs among 1,000 experiments for each 
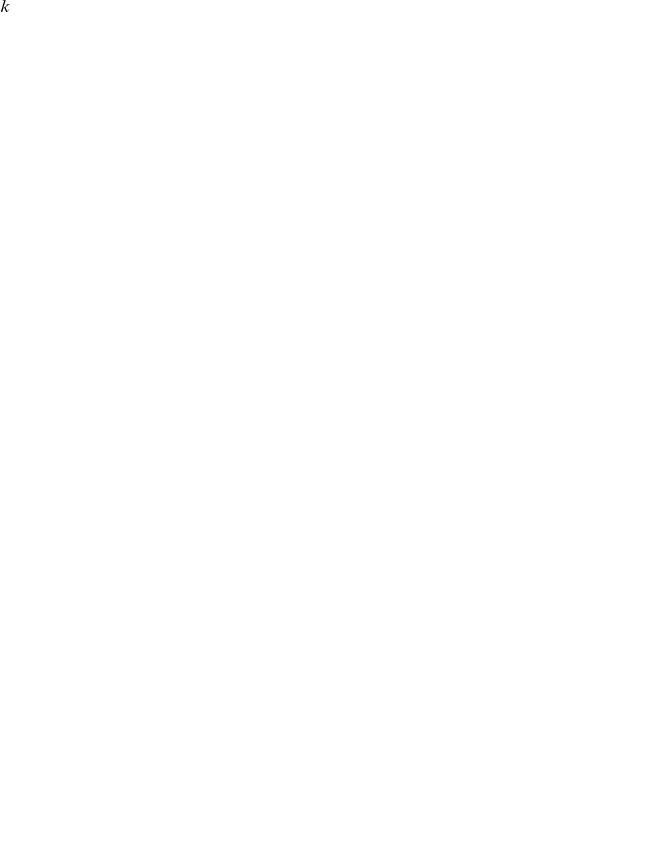
. In Table 5, we summarize the mean and standard deviation of 95% CIs from experiments of 20, 30 and 40 simulation runs. Based on these numbers, it is safe to say that a sensible decision is to repeat each simulation at least 30 times.

**Table 5:** Selected Simulation Properties.

Simulation Property	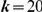	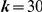	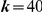
Day of the 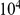 -th case	0.98 (0.13)	0.79 (0.08)	0.68 (0.05)
Day of the 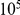 -th case	1.06 (0.14)	0.84 (0.08)	0.72 (0.05)
Day of the 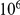 -th case	1.06 (0.15)	0.85 (0.09)	0.73 (0.06)
Day of the 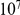 -th case	1.42 (0.18)	1.14 (0.11)	0.98 (0.07)
Number of infected people	2,890 (420)	2,330 (270)	2,000 (180)

The relationship between the number of simulation runs (
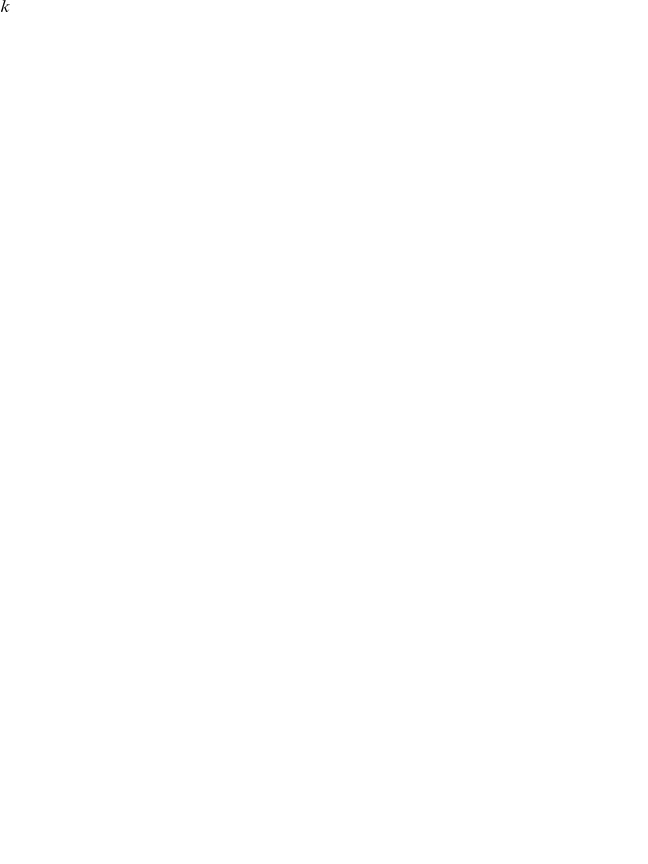
) and 95% CI for several simulation properties. The mean and standard deviation, in parentheses, of 95% CI per 1,000 groups of 
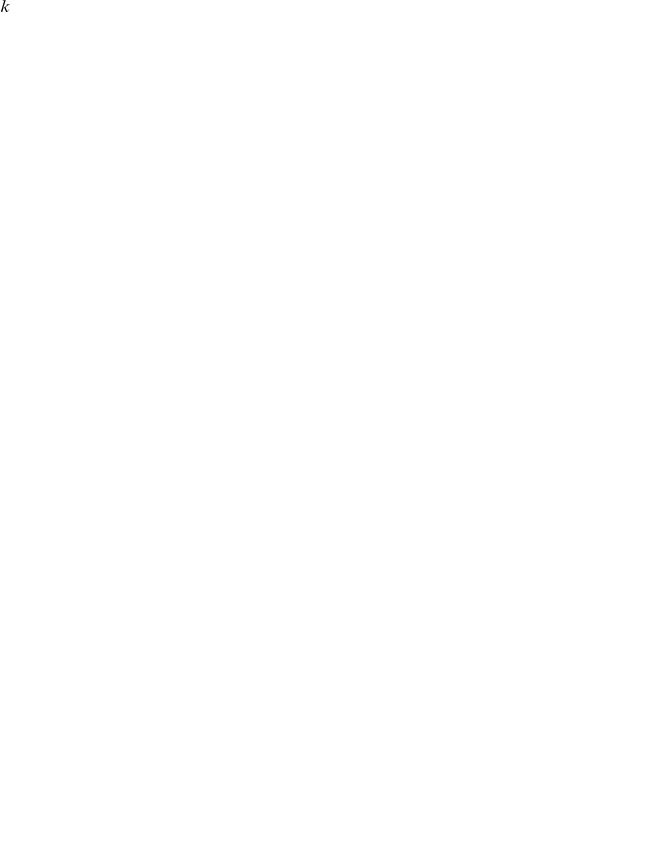
 simulation runs are shown.

### Practical use of efficient simulations

To demonstrate the practical use of the model, we simulate a severe flu pandemic, 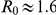, in Taiwan. We design two scenarios to best describe typical epidemic outbreaks: (1) An imported infectious case by seeding one index case in Taipei, which is at the northern end of the island and is densely populated with over 2.6 million people in the city and over 5 million in the greater metropolitan area; as the political, economic, and cultural center of the nation, Taipei is the most likely first stop for all international travelers. (2) An endemic outbreak by seeding one index case in a mid-latitude, less connected, remote farming town in Changhua county, which has 1.3 million residents and the highest concentration of chicken livestock in the country. We run each scenario 1,000 times. For the Taipei scenario, the single index case causes an outbreak in 513 out of 1,000 runs; while for the Changhua scenario, 543 runs result in outbreaks. We plot the averages of these outbreaks and observe that the epidemic progresses more rapidly from Taipei to other areas, resulting in a more synchronized epidemic; that is, the number of incidences is similar in quite distant locations during the middle part of the epidemic. In contrast, Changhua is less well connected, and the epidemic takes longer to spread to other parts of the population. Hence, the number of incidences in the mid-latitude area close to the seed is higher than in other areas. This results in a slightly slower epidemic (in terms of growth), but the peak is more pronounced for the Changhua scenario. These simulation runs illustrate the general principal that when epidemics fail to synchronize spatially, the overall incidence is less peaked. However, the results presented here do not describe local incidences of infection, which would be more “peaky”. The animation in Movie S1 (which is published as supporting information) demonstrates the spatial epidemiology of infectious disease for both scenarios. The simulation cases presented in this movie were selected to approximate, as closely as possible, the calculated average of all 500+ simulation runs for each scenario. We also prepared another movie (Movie S2, which is published as supporting information) by selecting simulation runs that were farther away from the average behavior of each scenario to show the unpredictable nature of the stochastic process.

In Movie S3 (which is published as supporting information), we use a different representation to demonstrate the county level spread of infectious disease, where each rectangular bar represents a county or major city in Taiwan; hence, their geographical relationships are also presented in these diagrams. The height of each bar indicates the number of new symptomatic cases daily; hence, we can easily observe the epidemic's critical level for each location.

**Figure 5:**
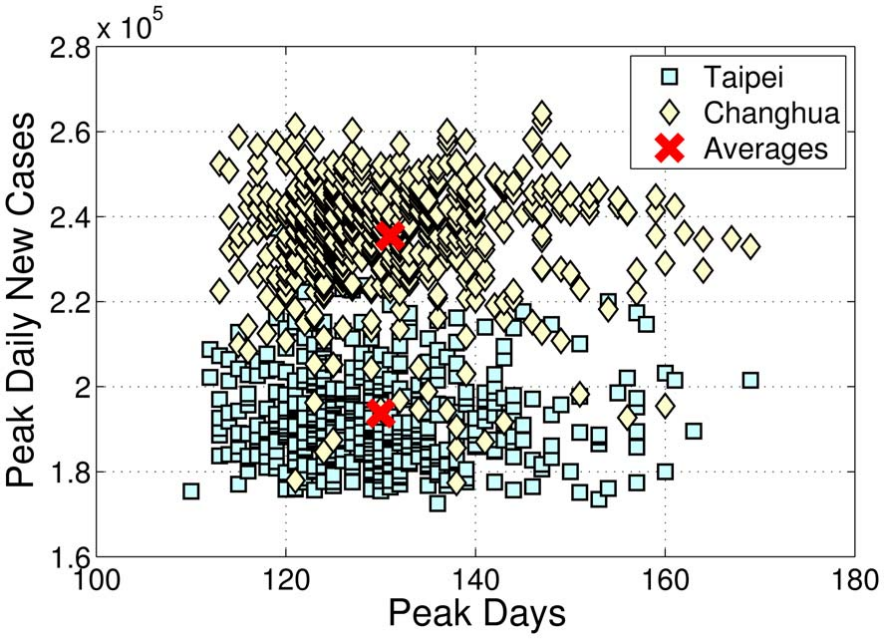
Simulation peaks distribution (with averages) for Taipei and Changhua scenarios when outbreaks occurs with one index case in 1,000 simulation runs. The 
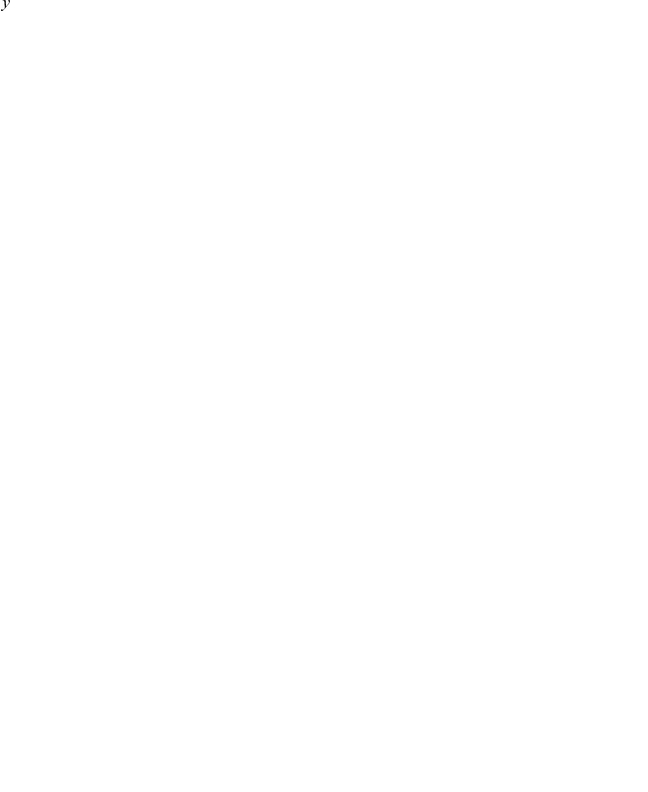
 axis shows the maximum daily new symptomatic cases of each simulated outbreaks (Taipei scenario, 95% CI 192,722–194,729; Changhua scenario, 95% CI 234,307–236,560), the 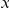 axis shows the day that peak occurs (Taipei scenario, 95% CI 129–131; Changhua scenario, 95% CI 130–132).

**Figure 6:**
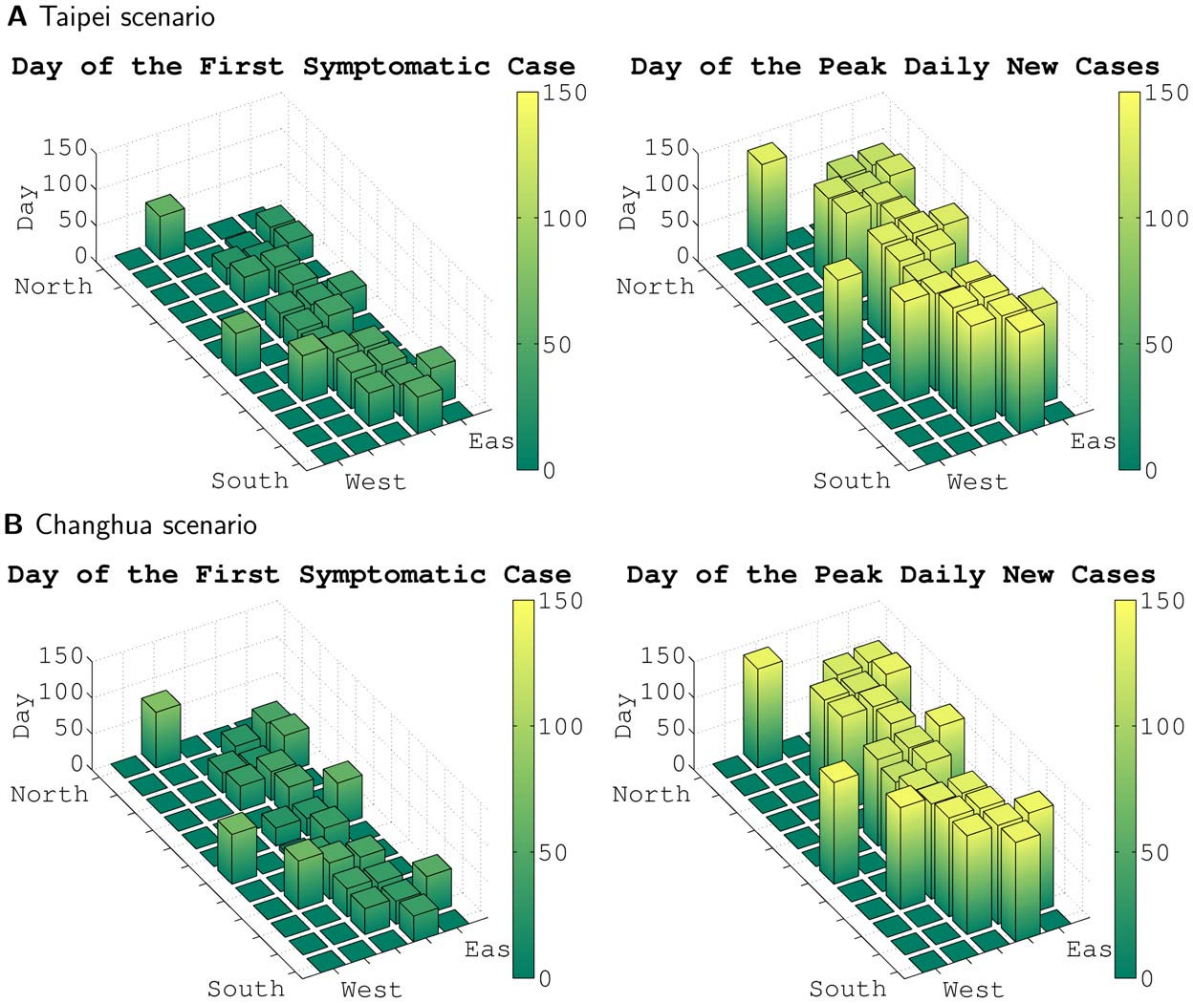
County level spatio-temporal spreading patterns for Taipei and Changhua scenarios.

If we use the peak of new cases and its date as an indicator of each outbreak and plot the distribution of all simulation runs for both scenarios, we find that although they are reasonably scattered in a disk area, the two disks have a non-trivial overlay (Figure 5). Such observations may not be possible if only a few simulations are conducted. Figure 6 shows the spatio-temporal spreading patterns for the Taipei and Changhua scenarios. In each figure, the whole island of Taiwan is plotted as a rectangle. The day that an area reports the first symptomatic case is plotted on the left; and the day that the peak occurs in an area is plotted on the right. We observe that Changhua has a less uniform spatio-temporal spreading pattern. The Taipei scenario exhibits more coordinated behavior.

## Discussion

We have described the application of a general re-sampling algorithm to a widely used spatial model of infectious disease transmission [8]. The resulting epidemic simulation tool achieves substantial speedups compared with our own implementation of a naive algorithm for the same model. Although derived independently, the resulting simulation algorithm is similar to those used to investigate the properties of the re-emergence of smallpox in the UK [18], and the pandemic influenza in Thailand [19], the United Kingdom and the United States [4].

We believe that further research on the underlying algorithms for the model presented here and similar models is warranted. For example, there are many ecological questions about the spatial properties of the current H1N1pdm — not least the need to explain the high degree of spatio-temporal variability observed on a continental scale. More generally, on any scale, improved computational efficiency of epidemic models, similar to that demonstrated here, will substantially increase their utility as tools for theoretical investigation.

## Supporting Information

Appendix S1 Supporting text with implementation details.
(0.16 MB PDF)

Movie S1 Visualization of typical spatio-temporal spreading patterns of an influenza epidemic in Taiwan with index case seeding in two distinct locales. The daily prevalence of symptomatic cases in each community is presented as an epidemic alert level on a logarithmic color scale, with red indicating the most critical situation when 3% or more of the population become symptomatic.
(11.02 MB AVI)

Movie S2 Spatio-temporal spreading patterns of a rare influenza epidemic in Taiwan with index case seeding in two distinct locales.
(11.04 MB AVI)

Movie S3 County level visualization of influenza epidemic simulations in Taiwan.
(7.28 MB AVI)
