# Supplementary material for: Efficient Simulation of the Spatial Transmission Dynamics of Influenza
Source: PLoS One. 2010 Nov 4;5(11):e13292. doi: 10.1371/journal.pone.0013292 (PMC2973967; doi:10.1371/journal.pone.0013292)
Supplement: Appendix S1 — Supporting text with implementation details. (0.16 MB PDF) [file pone.0013292.s001.pdf]

Appendix: Implementation Details

To construct an efficient influenza simulation system aiming at Taiwan population, we base our model on Taiwan regional geographic data, Taiwan Census 2000 data (http://www.stat.gov.tw) and develop an integrated three modules, Initialization, Simulation, and Visualization, system as briefly shown in Figure 1.

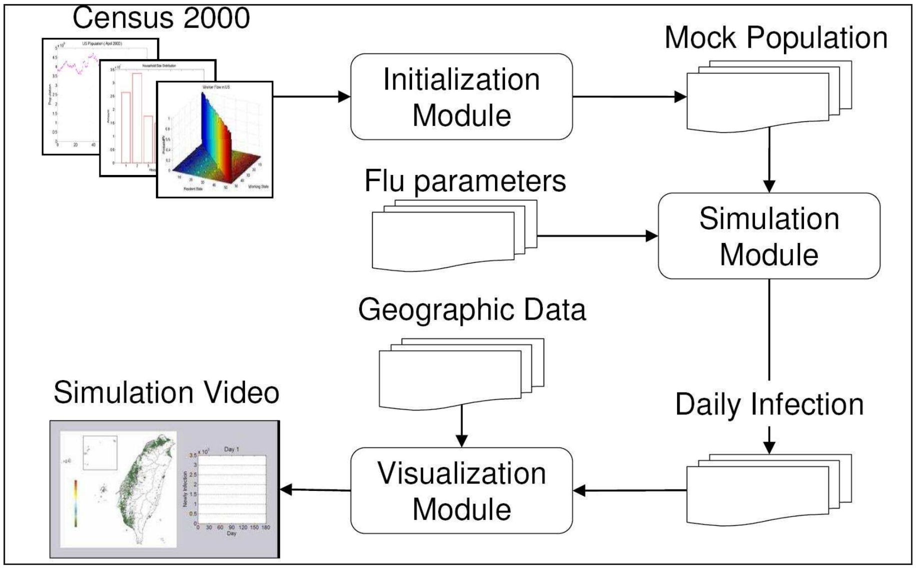

Figure 1. System architecture.

With the adaptation of various techniques in software optimization, not only is our system capable of evaluating diverse intervention strategies against influenza, the exceptionally improved efficiency also makes it possible to complete numerous simulations for any specific scenario within a reasonable time frame. The introduction to components of our system is as follows:

Initialization module

This module first reads a number of configuration files: Ps lists each town's id with the number of communities under that township, 369 entries for 369 towns in Taiwan; age.tbl lists the age and the number of people of that age, 101 entries for age 0 to 100, people with age 100+ years old are counted

as age 100; hst.tbl lists the family structures and the number of families of that structure, the family structure is defined as a five digit number, with the first digit (most significant digit) denotes the number of elders (65+ years old) living in that family, the second digits for the number of adults (30–64 years old), and so forth, please also note that the sum of all 5 digits will not exceed 7, since each household consists of one to seven people; wflow.tbl is the 369 by 369 matrix depicts the town-to-town worker flow as described in the previous paragraph.

The Initialization module will then go through all the towns listed in Ps, creates contact groups representing communities, neighborhoods, household clusters and households for each town. In this process, individual is stochastically created, assigned an age according to age distribution specification in age.tbl, and populated into a household according to respective age group and family structure distribution specification in hst.tbl. After the basic model of Taiwan population is generated with individual's age and residence information, the social contact groups for daytime activities are created with similar assumptions and process presented in the supporting information of this study [1]. Each individual is assigned to multiple social contact groups based on Taiwan demographics, including considerations for 5% unemployment rate and 7% dropout rate for school-age children. One final step is to adjust the size of each group if necessary. The average size for the final social contact groups are: ≈3 children for playgroups, ≈14 children for daycare centers, ≈106 children for elementary schools, ≈78 children for middle schools, ≈92 children for high schools, and ≈20 adults for work groups. Please note that work groups and school groups do not represent any actual workplace or school but rather the groups of age-specific people who share the same surrounding activities and make regular, relatively close contact that could result in transmission of flu virus. It is worth mentioning that the module takes less than a minute to generate a mock Taiwan population of 23 million people.

Mock population

Once the Initialization module generates a mock population, the social network (daily contact patterns) among individuals is fixed. Each individual belongs to one out of five age groups, in the mean time, he/she belongs to multiple contact groups according to his/her daytime and nighttime activities. To speed up the simulation, we pay special attention to memory access patterns so that a high hitting rate of cache memory can be maintained. The results of repeated and complicated floating-point computations are stored to avoid recomputing. The status of communities and contact groups, as well as the attributes,

which reflect each individual's current state, are carefully arranged to reduce memory consumption and access time. Grounded on such a computational social network model, the Simulation module is able to simulate the influenza epidemic effectively and efficiently.

Simulation module

Taking the mock population and influenza parameters as inputs, this module simulates the epidemic progression in Taiwan. We had designed several ways to introduce index cases to initiate the epidemic, one of the methods is that we assume international air travelers are the dominant source of influenza infectors, and we can proportionally seed index cases according to the travel patterns and the prevalence of the epidemic regions. In the accompanied examples, one individual is chosen at random as the index case at the first day of the simulation, they are seeded in (1) the heart of Taipei city, a popular shopping district which is the first stop of the majority of foreign and domestic tourists, (2) a small farming township in Changhua county, where they have the highest concentration of chicken livestock in the country. Both simulations cover 180 days, roughly the length of a Taiwan influenza season. In the Simulation module, we neglect to implement the occasional long distance travel, for Taiwan is a relatively small island, about 400 km long and 145 km wide. The distance of those occasional travel, be it for business or leisure, is unlikely to exceed the distance covered by the daily commuter travel for work. The worker flow is the dominant force for spreading the disease.

The module runs in cycles of two 12-hour periods (daytime and nighttime), during which we identify several contact groups. In five of these groups (households, household clusters, preschools, playgroups, schools, and work groups), relatively close contact regularly occurs. Additionally, neighborhoods and communities are for modeling unspecified groups, e.g., shopping malls, within which occasional contact occurs. Daytime contact occurs in all contact groups, while nighttime contact occurs only in households, household clusters, neighborhoods, and communities.

Conceptually, for each contact group we calculate the infection probability for its susceptible members according to the infectors within. After running through all contact groups, we flip a coin to decide if the susceptible individual is infected. Following the influenza model introduced in Section 2, influenza parameters from study [1], and the person-to-person contact shown in the mock population, the Simulation module outputs the number of newly infected individuals after every 24-hour simulation.

Containment and mitigation measures are scheduled events altering intended model parameters such

as epidemiological attributes or behavior patterns of some selected groups of population. Our simulation module covers most of the intervention strategies in various details, and is a useful tool for exploring the effects of any mitigation measure or assembly of joined strategies. Recent H1N1pdm presents an invaluable opportunity of verifying simulation authenticity against stats derived from conducting pandemic preparedness and response exercises. Our current work is focusing on the school closure strategies implemented in Taiwan during H1N1pdm. Several interesting scenarios regarding school closure strategies are also available in this study [2].

Visualization module

In order to make the results of the Simulation module self-explanatory, the Visualization module transforms these seemingly meaningless numbers into a motion picture, which records the daily new cases for each local government as the influenza epidemic progresses. The color of the dot varies from green to red indicating the severity of the flu epidemic from safe to critical.

Computational Resources Requirement

To benchmark the computational resources requirement for our implementation, we perform baseline simulations with population of 287 million people. A typical production run ($R_0 \approx 2.0$) takes about 30 minutes to complete, and requires 32 GB memory on a server with dual Intel Xeon W5580, quad-cores, 3.20 GHz CPUs, as opposed to 256 Intel Xeon 2.4 GHz CPUs cluster, 8–12 hours in the previous study [1].

References

1. Germann TC, Kadau K, Longini IM, Macken CA (2006) Mitigation strategies for pandemic influenza in the United States. PNAS 103: 5935-5940.

2. Lee BY, Brown ST, Cooley P, Potter MA, Wheaton WD, et al. (2010) Simulating school closure strategies to mitigate an influenza epidemic. Journal of Public Health Management Practice 16: 252-261.
